# Differential Effects of Temperature Extremes on Hospital Admission Rates for Respiratory Disease between Indigenous and Non-Indigenous Australians in the Northern Territory

**DOI:** 10.3390/ijerph121214988

**Published:** 2015-12-03

**Authors:** Donna Green, Hilary Bambrick, Peter Tait, James Goldie, Rosalie Schultz, Leanne Webb, Lisa Alexander, Andrew Pitman

**Affiliations:** 1 Climate Change Research Centre, University of New South Wales, Sydney 2052, Australia; j.goldie@unsw.edu.au (J.G.); r.schultz@student.unsw.edu.au (R.S.); Leanne.Webb@csiro.au (L.W.); l.alexander@unsw.edu.au (L.A.); a.pitman@unsw.edu.au (A.P.); 2 ARC Centre of Excellence for Climate System Science, University of New South Wales, Sydney 2052, Australia; 3 Centre for Health Research, School of Medicine, Western Sydney University, Sydney 2150, Australia; H.Bambrick@westernsydney.edu.au; 4 Public Health Association of Australia, Canberra 2605, Australia; aspetert@bigpond.com

**Keywords:** indigenous health, temperature extremes, respiratory health, climate zones, Aboriginal Australia, disproportionate impacts, climate change

## Abstract

The health gap between Indigenous and non-Indigenous Australians may be exacerbated by climate change if temperature extremes have disproportionate adverse effects on Indigenous people. To explore this issue, we analysed the effect of temperature extremes on hospital admissions for respiratory diseases, stratified by age, Indigenous status and sex, for people living in two different climates zones in the Northern Territory during the period 1993–2011. We examined admissions for both acute and chronic respiratory diagnoses, controlling for day of the week and seasonality variables. Our analysis showed that: (1) overall, Indigenous hospital admission rates far exceeded non-Indigenous admission rates for acute and chronic diagnoses, and Top End climate zone admission rates exceeded Central Australia climate zone admission rates; (2) extreme cold and hot temperatures were associated with inconsistent changes in admission rates for acute respiratory disease in Indigenous and non-Indigenous children and older adults; and (3) no response to cold or hot temperature extremes was found for chronic respiratory diagnoses. These findings support our two hypotheses, that extreme hot and cold temperatures have a different effect on hospitalisations for respiratory disease between Indigenous and non-Indigenous people, and that these health risks vary between the different climate zones. We did not, however, find that there were differing responses to temperature extremes in the two populations, suggesting that any increased vulnerability to climate change in the Indigenous population of the Northern Territory arises from an increased underlying risk to respiratory disease and an already greater existing health burden.

## 1. Introduction

Humans can acclimatise to living in extreme temperatures. From the Afar people who mine salt in the Danakil Depression, Ethiopia, where temperatures regularly exceed 50 °C [1], to the Yakut hunters who experience mean winter temperatures of minus 48 °C in Verkhoyansk, Russia [2], the human body is vastly adaptable to ambient temperatures.

However, as the death toll from heatwaves in Europe, North America and Russia has shown [3,4,5], the inability of some population subgroups, such as the elderly, to cope with heat extremes suggests that human capacity to adapt to heat is limited, and there is a limit to which even healthy humans can acclimatise [6]. Further, external factors, such as air pollution, pre-existing disease and social conditions, can limit that adaptability [7,8,9,10].

In Australia, there is already abundant evidence of shorter life expectancy for Aboriginal and Torres Strait Islanders, who make up the Indigenous population, compared with non-Indigenous Australians [11]. Reasons for this disparity are well documented [12], as are attempts by governments to address it through targeted policy interventions [13,14]. Leading causes of excess mortality among Indigenous Australians include circulatory disease (25 per cent) and respiratory disease (9.5 per cent) [11].

An exploration of whether there is a disproportionate greater impact on health from climate sensitive diseases among Indigenous people is important due to established links between heart and respiratory diseases and ambient climate in the general population [15,16,17]. The greater respiratory disease burden experienced by Indigenous people compared with non-Indigenous people [11], suggests that unless proactive steps are taken to reduce the indirect impacts on life expectancy of recent and future climate change, the current gap is likely to increase.

In order to explore this problem, and to estimate any disproportionate impacts found relating to Indigenous status, we tested two hypotheses. The first was that extreme hot and cold temperatures have a different effect on hospitalisations for respiratory disease between Indigenous and non-Indigenous people, and the second was that the impact of temperature on these respiratory diseases varies by climate zone.

Developing a better understanding of the relationships between temperature and hospitalisations among vulnerable populations can be used to inform public health policy to proactively prepare health systems for climate change and enable tailored, local level adaptation measures. This knowledge will ensure that support is focused on subpopulations most likely to benefit from action to respond to climate change [18,19].

## 2. Background

There is clear evidence that temperature extremes can have significant impacts on human health in Australia [15,20]. Studies show that the impacts are not distributed equally, with some subpopulations identified as particularly vulnerable [21,22,23]. While many of these findings reflect well-known human health impact studies reported outside Australia [24], the disparity relating to Indigenous status has limited international precedent [25,26].

It is anticipated that climate change will exacerbate existing health disparities between Australia’s Indigenous and non-Indigenous populations [27]. This assumption is supported by two quantitative studies that assessed temperature sensitivity among Indigenous people living in the Northern Territory [28,29]. The first study found that while hotter minimum temperatures were associated with an increased risk of hospitalisation for Indigenous people, particularly overweight individuals and males generally, colder minimum temperatures were associated with an increased risk of hospitalisation for women. Both cold and hot temperatures were associated with an increased risk of hospitalisation for older Indigenous people. The second study analysed the link between climate extremes and cardiovascular disease in admissions to Northern Territory hospitals during 1993–2011. It found a significant relationship between climate extremes and hospitalisations for cardiovascular disease, which varied by Indigenous status, sex and age.

After cardiovascular diseases, respiratory diseases are the second most frequent cause of hospital admission in Australia, and disproportionately affect children and the elderly. At the national level, Indigenous Australians have a 2.7 times higher rate of hospital admission for respiratory diagnosis than non-Indigenous Australians. Leading respiratory diagnoses for Indigenous hospital admissions are pneumonia, chronic respiratory disorders and asthma [30].

Respiratory diseases can be either acute or chronic, with the former responsible for most hospital admissions. Acute respiratory infections caused by viruses or bacteria affect either the lower (below the vocal cords) or upper respiratory tract. The most common (upper respiratory) infections are colds, acute sinusitis, acute pharyngitis and acute tonsillitis. Lower respiratory diseases include pneumonia, bronchopneumonia, acute bronchitis and bronchiolitis, which, although less common than upper respiratory infections, are generally more serious and trigger more hospital admissions. Chronic respiratory diseases include asthma and chronic obstructive pulmonary diseases (COPD), comprising chronic bronchitis, emphysema and bronchiectasis [30]. Chronic bronchitis and emphysema are caused principally by smoking and are exacerbated by a number of factors, including infections and air pollution [30].

Given the links between respiratory disease and climate [17,31], and the projected changes to climate, this issue merits further investigation. This analysis is especially important in relation to the likely impact on the Indigenous population specifically, due to the existing health disparity between Indigenous and non-Indigenous people. 

**Figure 1:**
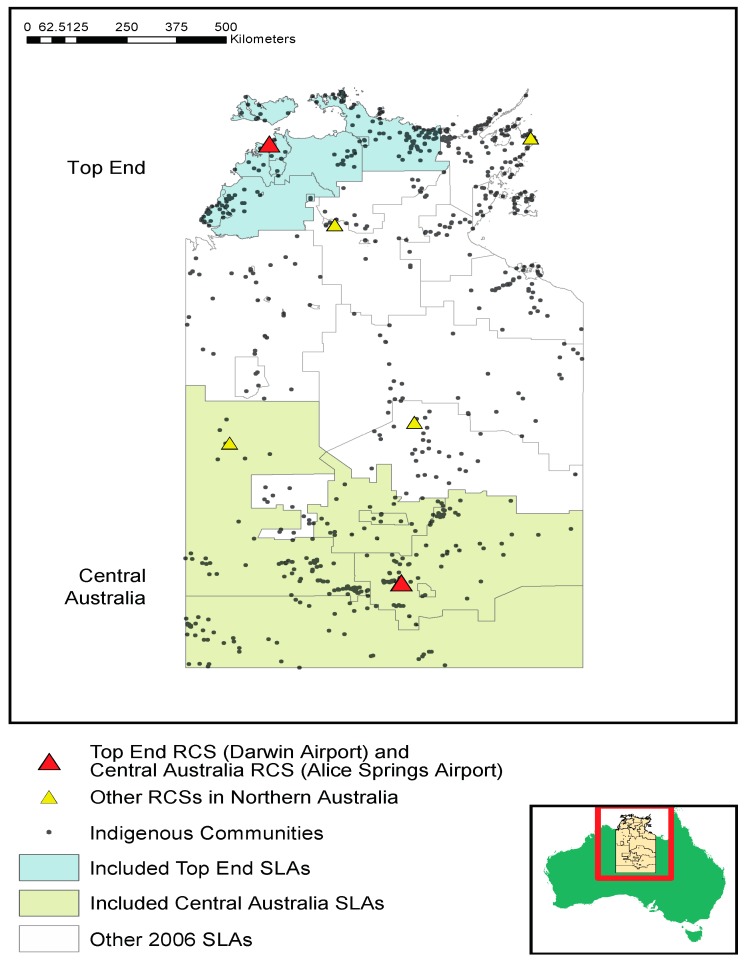
Location of communities, climate zones and Reference Climate Stations (RCS).

The Northern Territory consists of almost a third of the continent’s north and includes the coastal city of Darwin and the inland city of Alice Springs. Almost one-third of the population in the Northern Territory is Indigenous—an order of magnitude higher than that of any other Australian State or Territory [30]. The Northern Territory covers 1,349,129 km^2^, the majority of which is falls into one of two major climate zones: tropical and semi-arid. The majority of the population in the Northern Territory, and hence the majority of hospital admissions, reside within these climate zones in Australian Bureau of Statistics’ defined Statistical Local Areas (SLA) (Figure 1).

**Figure 2:**
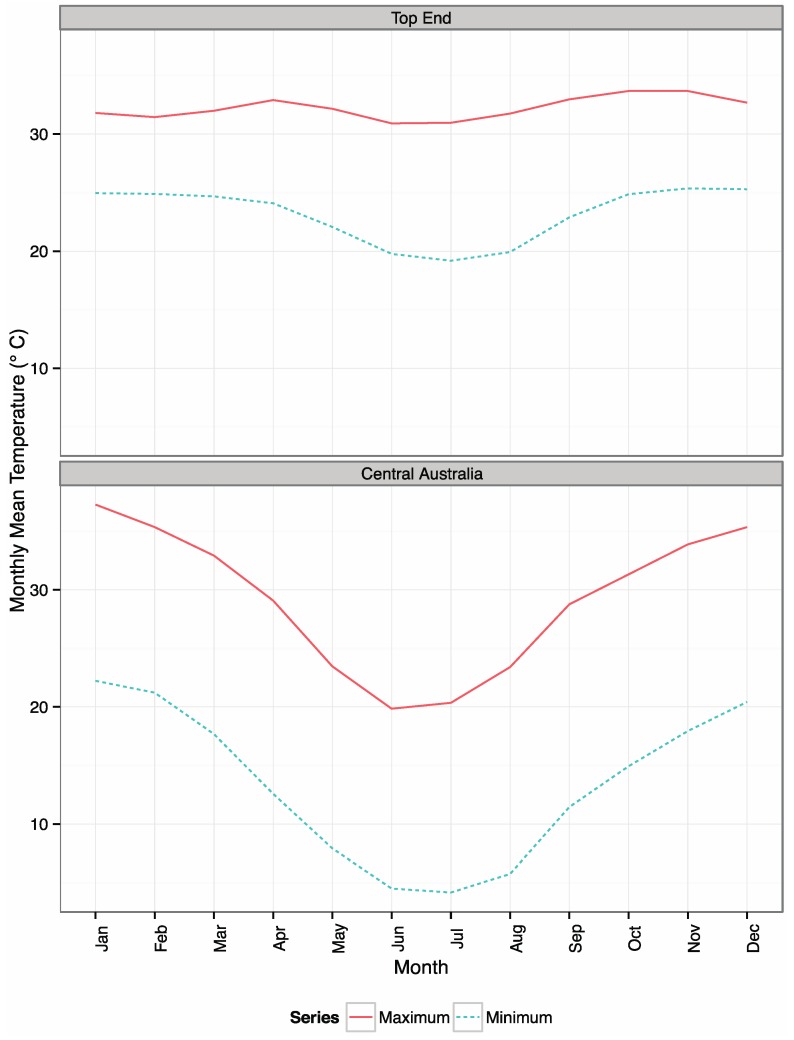
Mean monthly maximum and minimum temperatures for Darwin Airport RCS and Alice Springs Airport RCS, representing the “Top End” and “Central Australia” climate zones respectively.

The northern climate zone, the “Top End”, has a variable tropical climate, with high humidity and two seasons: the wet season, spanning November to April; and the dry season, from May to October. The southern climate zone, “Central Australia”, where Alice Springs is located, is semi-arid, with a highly variable, hot summer/cold winter climate. The mean monthly maximum and minimum temperatures of these two climate zones are shown in Figure 2.

## 3. Methods

### 3.1. Hospital Admission Data

Admissions data for the period 1993 to 2011 were obtained from all public hospitals in the Northern Territory: the two major hospitals, Royal Darwin and Alice Springs, and three smaller regional hospitals, Gove, Katherine and Tennant Creek. Two geographically determined population groups were identified for analysis, based on place of residence, as recorded in hospital admission data in relation to the climate zones identified in Figure 1. Due to very low population densities which limited the building of stable population estimates between censuses, and shifting SLA boundaries, several regions were not included in the analysis. The two regions selected for the analysis are shaded in Figure 1; other regions that were not included are unshaded. The ambient temperature at the time of patient’s admission was based on their usual place of residence as defined by the Australian Bureau of Statistics (ABS) SLA, a geographic unit consisting of approximately 10,000 people.

Variables analysed were: admission date, age, sex, Indigenous status, acute and chronic respiratory diagnosis codes, and patients’ place of residence using 2006 SLA borders. International Classification of Diseases (ICD) codes were used to select admissions to include in the analyses. Over the time period, there were about 19,000 hospital admissions with the primary diagnosis for the disease groups of interest for acute respiratory and for chronic respiratory from ICD-9 and ICD-10 (the selected ICD codes for each grouping are provided in supplementary material); the geographically determined groups were stratified by their diagnosis groups (we excluded asthma because it has a large number of triggers, and there would have been insufficient statistical power to separate a temperature effect from these triggers).

These geographically determined groups were then stratified by age group, Indigenous/non-Indigenous status and sex. Age groups were defined based on the age distribution of admission counts: acute admissions demonstrated a U-shaped distribution, with the lowest counts in the 10–34 year old bracket, and chronic admissions occurred mostly in people over 35 years of age. Hence, the age groups 0–9 years, 10–34 years and 35+ years were selected, a factor also influencing the choice of age brackets was the smaller number of Indigenous population at older ages and each age group was analysed separately.

Age-adjusted admission rates for each age group in each of the two climate zones were calculated by dividing the number of admissions each day by an estimate of the regional population, then multiplying this figure by each group’s fraction of a standard Australian population. Daily population counts for use as the denominator were estimated by linearly interpolating Australian Census counts by age, sex and Indigenous status for the statistical regions of the RCS catchments for 1991, 1996, 2001, 2006 and 2011 [32]. Because the borders of SLAs change from year to year, SLAs from Census years other than 2006 were allocated to catchments in the same way. In cases where Indigenous community data indicated that most of the population lived within the catchment, that region was included.

### 3.2. Climate Data

Daily temperature records for the study area were available from six weather stations belonging to the RCS network. Darwin Airport was selected to represent the “Top End” climate zone, and Alice Springs Airport to represent the “Central Australia” climate zone. While each of the climate zones cover large areas, the two selected weather stations are located where the populations for each region are concentrated and they therefore give an indication of the outdoor ambient temperatures that residents of each region would have been exposed to. For each of the two datasets, the ≤10th percentile (“cold extreme”) and ≥90th percentile (“hot extreme”) were calculated from daily minimum temperature (Tmin, °C), and daily maximum temperature (Tmax, °C). Days (defined 9 am to 9 am) in the time series that were colder than the cold extreme are defined as “cold days”; those hotter than the hot extreme are defined as “hot days”. Temperature effects in this analysis are represented by the ratio of admission rates on the 10 per cent of cold days to the 90 per cent of not cold days, and on the 10 per cent of hot days to the 90 per cent of not hot days.

Daily hospital admissions were extracted from the hospital admissions dataset using the selected ICD codes for acute and chronic respiratory diseases, and these data were then merged with the weather records from the relevant Reference Climate Station (RCS). In this way, each admission was identified as having occurred either on or subsequent to, a cold day or hot day in relation to a single temperature point, rather than a heatwave.

Poisson loglinear models were built for each group in the two populations, as stratified by diagnosis (acute or chronic), age group, Indigenous/non-Indigenous status and sex. The models’ response variable was the daily admission count. Each model used one binary predictor indicating each day as either a cold day or a hot day, as defined by Tmax or Tmin; the coefficient calculated for this predictor in each model was exponentiated, along with 95 per cent confidence intervals, to produce admission rate ratios for the “cold” days in the time series in relation to the “not cold” days, and for the “hot” days to “not hot” days. These rate ratios indicate the responses to temperature extremes, with resulting rate ratios greater than one associated with increased rates, and rate ratios less than one with decreased rates.

Other predictors in the models included a day of the week factor, a natural spline of time with four degrees of freedom for each year of the time series to account for seasonality [33], and a logged daily offset of estimated population, as discussed in Section 3.1. Since respiratory conditions display a lag effect for morbidity [34], we assessed admission rates on the day of extreme weather, and for lags of one, two, three, five, seven and ten days.

## 4. Results

### 4.1. Admission Rates by Indigenous Status

**Figure 3:**
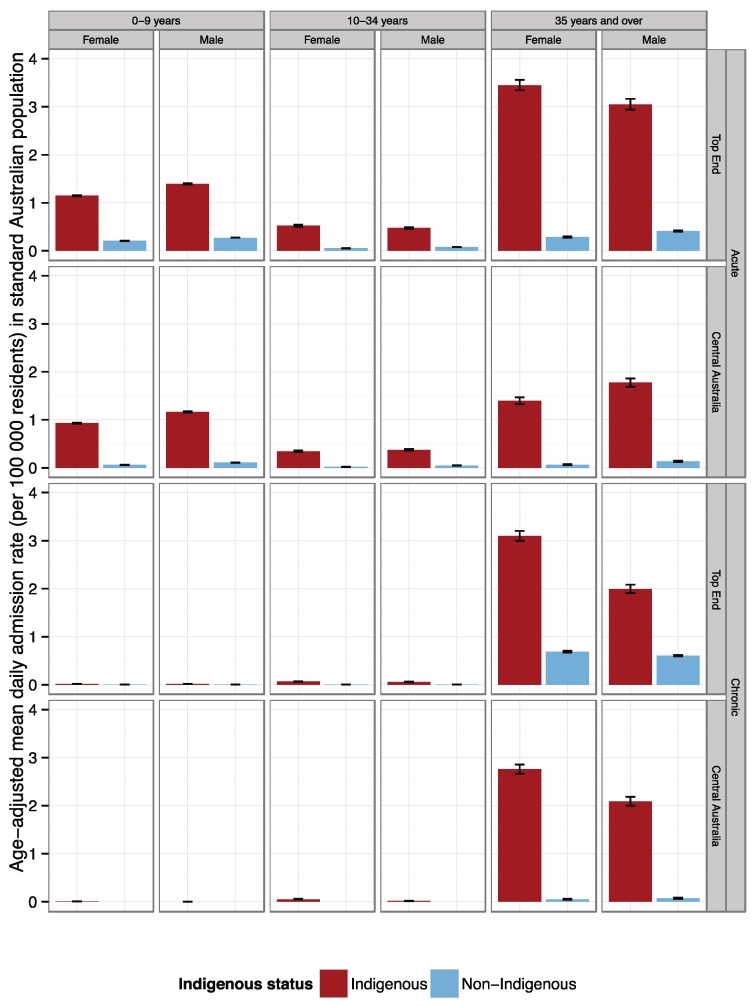
Age-adjusted daily admission rates, per 100 000 residents, for acute and chronic respiratory conditions, for the Top End climate zone and Central Australia climate zone by Indigenous/non-Indigenous status, age and sex.

Age-adjusted admission rates were higher for the Indigenous populations in both climate regions examined and for both acute and chronic diagnoses (Figure 3).

Generally, admission rates for acute and chronic conditions were higher for people living in the Top End climate zone compared to the Central Australia climate zone. Stratified by disease class, admission rates for people with acute respiratory conditions were almost twice as high in the Top End climate zone as those for people living in the Central Australia climate zone.

Within these acute admissions, there were peaks in the 0–9 and 35 year and over age groups with males generally showing higher admission rates than females. For chronic respiratory diseases, in the 35 year and over age group, Top End climate zone rates were similar to Central Australia climate zone rates for Indigenous people, but substantially higher for non-Indigenous people.

### 4.2. Sensitivity of Respiratory Disease Admission Rate to Temperature

Our analysis found that admissions sensitivity to temperature extremes for acute respiratory diseases varied by temperature, region, Indigenous status and sex. We present separate results for each of these groups for acute and chronic conditions.

#### 4.2.1. Acute Respiratory Conditions

**Figure 4:**
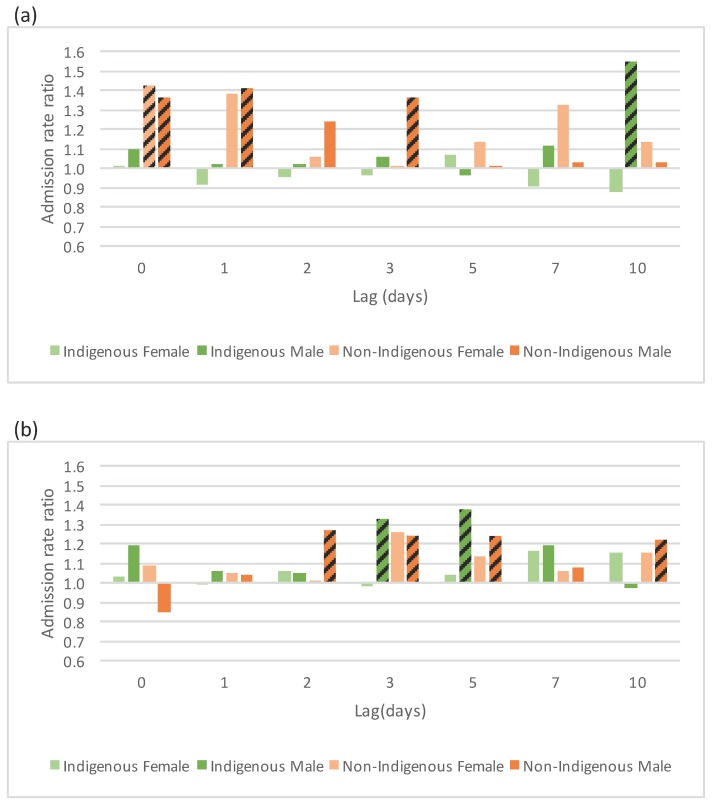

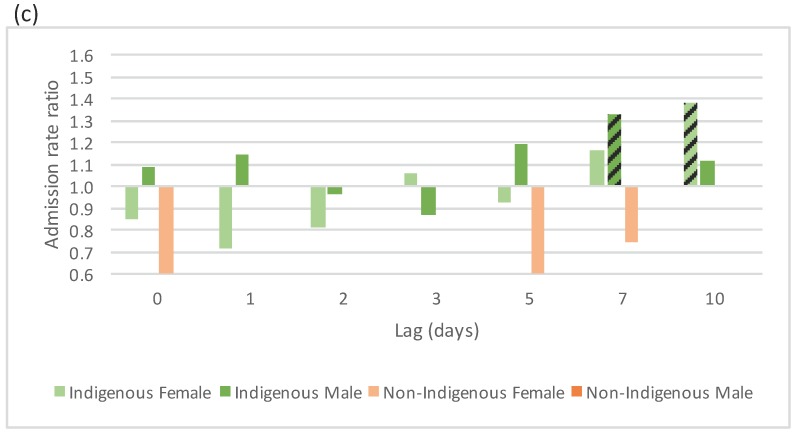
Selected cold effects on acute respiratory admissions, expressed as rate ratios, by Indigenous status and sex over lags of 0 to 10 days. (**a**) Cold effects, defined by Tmin, on residents aged 0–9 in the Top End climate zone; (**b**) cold effects, defined by Tmax, on residents aged 35+ in the Top End climate zone; (**c**) cold effects, defined by Tmin, on residents aged 0–9 in the Central Australia climate zone. Effects that are statistically significant at the five per cent significance level are highlighted with a diagonal line.

In the Top End climate zone’s cold days, no clear pattern was found in acute respiratory conditions for Indigenous children, while there was an increase in non-Indigenous child admissions among both sexes. For males, this increase extended out to a lag of three days (Figure 4a). In the over 35 age group, both Indigenous and non-Indigenous males (but not females) had increased admissions after cold days at longer lags (Figure 4b). Associated with cold days in Central Australia climate zone, there was an increase in acute respiratory admissions for children at longer lags (Figure 4c). No changes were found for the 10 to 34 years group.

**Figure 5:**
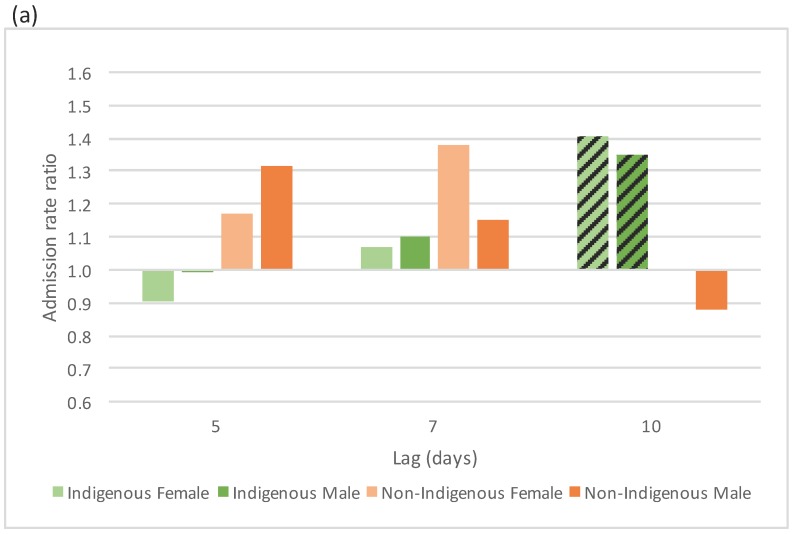

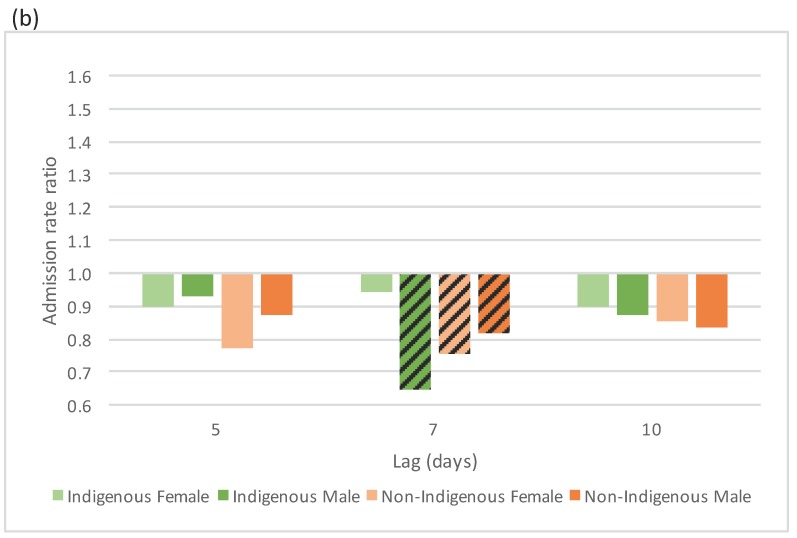
Selected hot effects on acute respiratory admissions, expressed as rate ratios, by Indigenous status and sex over lags of 5 to 10 days. (**a**) Hot effects defined by Tmin, on residents aged 0–9 in the Central Australia climate zone; (**b**) hot effects, defined by Tmax, on residents aged 35+ in the Top End climate zone. Effects that are statistically significant at the five per cent significance level are highlighted with a diagonal line.

No consistent pattern of increased admissions due to hot days was observed, but there were isolated significant results which may be the result of multiple testing. There were increases in admissions in the Central Australian climate zone among Indigenous children at a 10 day lag after a hot day defined by Tmin (Figure 5a). In the Top End climate zone there were reduced admissions among Indigenous males and non-Indigenous males and females aged over 35 years at day seven after a hot day defined by Tmax (Figure 5b).

#### 4.2.2. Chronic Respiratory Conditions

Due to the small numbers of children and younger adults admitted with a chronic respiratory condition, only the 35 years and over age group was analysed. There were no increases or decreased in admissions observed for either heat or cold extremes in either climate zone.

## 5. Discussion

The acute respiratory conditions included in this study were infections, such as pneumonia, while the chronic respiratory conditions that lead to hospital admission in this study are mostly due to exacerbation of chronic lung disease following an infection. Because of these different groups, divergent acute and chronic condition patterns emerged: overall, acute disease was seen at higher rates in children and older adults (Figure 2), while chronic disease was, as expected, seen almost exclusively in older adults. Pneumonia is associated with extremes of heat [35,36] and chronic obstructive pulmonary disease with extremes of cold. Studies of heat-related respiratory admissions in California [36] and Europe [37] both found that heat increased respiratory admissions, although the causal mechanisms were not well understood.

Many diseases and all-cause mortality show a U-shape distribution in relation to temperature, with cold/winter mortality greater than summer, particularly for respiratory diseases [38,39,40]. If only the association with heat (or heat-humidity) or cold is studied, however, a linear relationship is found at each extreme [31]. Two studies have recently reviewed the literature on the effects of heat and cold on mortality and morbidity [17,31], and one other study found temperature and humidity effects on Darwin residents [41]. A wide range of responses to temperature and humidity was reported, with all studies reporting increases in more extreme temperature conditions.

### 5.1. Indigenous and Non-Indigenous Admission Rates

The overall higher rates of hospital admissions for acute and chronic respiratory disease in Indigenous people of all ages and regions support what was expected from the literature [30], and highlights the need for on-going efforts to reduce the health gap between non-Indigenous and Indigenous people. The pattern of higher rates of acute admissions in children and the elderly, and small number of admissions for chronic conditions among children, were also expected. Rates of admission for acute respiratory disease for both Indigenous and non-Indigenous people at all ages and both sexes were higher in the Top End climate zone than in Central Australia climate zone, with the admission rate almost double for adults over 35 years. Admission rates for acute respiratory conditions for females over 35 exceeded those for males in Top End climate zone. For chronic respiratory conditions, while admission rates were similar for Indigenous people in each climate zone, rates for non-Indigenous people were much higher in the Top End climate zone. This reflects the patterns of admissions found in the age and sex analyses.

### 5.2. Sensitivity of Acute Respiratory Admission Rate to Temperature Extremes

#### 5.2.1. Responses to Cold

While the cold effect literature looks more at hypothermia, some mild effects are seen with cold exposure even when core body temperature is maintained [42]. Colder temperatures drive broncho-constriction, *i.e.*, narrowing of the airways [31], and reduce ciliary activity, which in turn reduces clearance of increased secretions. In general, with reduced temperatures and reduced humidity, we would expect this increase in acute respiratory infections [34,43].

Our results show increases in admissions among non-Indigenous children in the Top End climate zone on and shortly after cold mornings, as well as in Indigenous children in Central Australia climate zone at longer lags. While these results concur with the literature, we cannot explain the absence of corresponding effects among Indigenous children in the Top End climate zone and non-Indigenous children in Central Australia climate zone.

Positive associations seen among children in this study in the Top End climate zone between admissions at cold extremes fits the expected pattern of response to cold temperature extremes where, for example, Guo *et al.* [28], Wang *et al.* [44] found an increase in admissions with cold.

Similarly, men over 35 in the Top End climate zone showed an expected increase in admissions after cold afternoons—shortly after in Indigenous men, and at broader timescales, in non-Indigenous men—but this effect was not seen in women in this age group.

#### 5.2.2. Responses to Heat

Different patterns were seen in the Top End climate zone and Central Australia climate zone for acute respiratory admissions in response to heat. In the Top End climate zone we found no heat effect in children. The reduction in admissions for adults 35 years and over except among Indigenous women is difficult to explain. It is unlikely to be a statistical anomaly because it occurs across ethnicity and gender groups for hot afternoons with a lag of seven days. It may be related to factors other than temperature, such as humidity with rain, or relative cooling.

The increase in admissions among Indigenous children in the Central Australia climate zone ten days after a hot night might be a manifestation of pneumonia, the acute respiratory condition most likely to result in an admission for Indigenous children. The absence of any association in the non-Indigenous population may be a marker of the health differential, and possibly social and economic factors, ranging from reduced crowding to fewer introduced infections. We were not able to record access to air-conditioned environments, and so we may have missed possible associations in relation to cooled home environments.

### 5.3. Sensitivity of Chronic Respiratory Admission Rate to Temperature Extremes

There are several possible explanations for a lack of statistically significant changes in chronic respiratory condition admissions with temperature extremes. Admissions for exacerbations of chronic lung diseases may be captured in the acute disease diagnoses. Absolute numbers may be inadequate for variations to reach statistical significance—a lack of statistical power. However, from the data examined here, it appears that temperature extremes have no impact on chronic respiratory illness in either Indigenous or non-Indigenous adults. Furthermore, heatwaves, as extended periods of increased heat may have effects on hospital admissions that our data examining single hot days did not detect.

## 6. Limitations

Exposure to particulates from seasonal bushfires may contribute to admissions for both acute and chronic conditions during the Top End climate zone dry season [45,46,47] but we were not able to test for this, nor were we able to look at other respiratory irritants, such as ozone. Correction of data for seasonality may have controlled for the effects of exposure to bushfire smoke, which is a seasonal event. While Hanigan *et al.* [46] found that dry, cool-season bushfire smoke did affect respiratory admissions on the same day and, for Indigenous people, with a three day lag, that study observed no temperature or relative humidity effects. Our Top End climate zone findings for an increase in lagged admission rates for acute respiratory conditions may reflect this.

In order to assess climate impacts on health, we had to assume that patients were exposed to the temperature and humidity conditions of their usual region of residence immediately before their admission to hospital. We believe this assumption was reasonable, because even if patients were not in their usual place of residence when they became sick, but in a nearby location with friends or family, there was a high chance that they had experienced the same, or similar, climate extremes, due to the similarities in climate across the regions under analysis and the geographic concentrations of populations. We also did not analyse the role of barometric pressure, which has been found to have an association with COPD [48].

Hospital admissions data are limited by the admission criteria and data collection procedures. For example, Indigenous people may defer admission until they are severely ill after failed primary care or traditional treatment; alternatively, they may be admitted to hospital with less severe respiratory disease because of complex co-morbidity, living in remote regions or lack of social services and support outside of hospital. Since we only included admissions where the primary diagnosis was respiratory, admissions where a respiratory condition was not the primary diagnosis were not included. This is likely to affect Indigenous people disproportionately because of the high levels of co-morbidity [49].

Indigenous status may be incompletely identified in hospital admission data, which would potentially dilute any differences observed between the Indigenous and non-Indigenous populations. This is because recording of Indigenous status in Northern Territory hospital data may have been affected by misclassification, most likely under-recording, of Indigenous status during the earlier years of this study. Recording of indigenous status is now recognised as adequately complete and accurate in Northern Territory hospitals [49].

Our choice of 35 years is a threshold to define an older age group age in this population recognises the young demography of Aboriginal people. The median age of Aboriginal people in the Northern Territory is 23.8 years, and only 3.4 per cent are over 65 years [50].

Our datasets did not include primary health care service treatments, which provide the majority of care for both acute and chronic respiratory disease [30]. However, this is likely to be the case regardless of the presence of a temperature extreme, so it should not bias the results. Additional cases of respiratory disease may have occurred in those who died of complications without confirmation of a primary respiratory disease diagnosis, but this would not be biased with regard to temperature extremes either. We note that patients who were transferred from a regional hospital to Darwin or Alice Springs Hospital for specialist treatment would have been counted as an admission in both hospitals but this is unlikely affected by temperature extremes.

Potential confounders and other potential contributing factors which we were not able to take into account include socio-economic status; tobacco, drug or alcohol use; co-morbidities including diabetes, heart disease and obesity; pollen and particulate levels exposure relating to windborne dust and smoke; and varying access to care. Overall lower socio-economic status of Aboriginal people, leading to less use of air-conditioning and higher rates of co-morbidity, may have increased the observed association of extreme weather events with hospital admission for this group. While such hospital admission does not necessarily indicate worse effect of temperature extremes on Indigenous people, it is an important consideration in service planning.

Finally, we note that the selected five percent significance threshold and the large number of population analyses undertaken mean that some significant results may have occurred by chance. The most robust observations are therefore those that exhibit some consistency between different strata.

## 7. Conclusions

Extreme ambient temperatures were more likely to be associated with increased hospital admission rates for acute respiratory disease among Indigenous than non-Indigenous people in the Northern Territory. The associations between temperature and admissions for respiratory disease differed across the two climatic zones we tested, suggesting some degree of acclimatisation or different triggers for disease and admission to hospital.

We therefore conclude that the health risks associated with a changing climate are unlikely to be uniform between Indigenous and non-Indigenous people or across different climate zones. Instead, the health risks associated with a changing climate may have more to do with underlying risk than with differential response to temperature extremes. The implications of these findings of changed admission rates in a warming climate need to be assessed more fully. This should enable the health impacts of climate change to be reduced, particularly on increasing inequity [51]. Efforts to close the gap between the health of Indigenous and non-Indigenous Australians will need to overcome the additional disadvantage that climate change will increasingly impose.
